# 24-Epibrassinolide Facilitates Adventitious Root Formation by Coordinating Cell-Wall Polyamine Oxidase- and Plasma Membrane Respiratory Burst Oxidase Homologue-Derived Reactive Oxygen Species in *Capsicum annuum* L.

**DOI:** 10.3390/antiox12071451

**Published:** 2023-07-19

**Authors:** Zhengyang Wen, Zhifeng Chen, Xinyan Liu, Jingbo Sun, Feng Zhang, Mengxia Zhang, Chunjuan Dong

**Affiliations:** 1 State Key Laboratory of Vegetable Biobreeding, Institute of Vegetables and Flowers, Chinese Academy of Agricultural Sciences, Beijing 100081, China; 2 College of Biology and Agricultural Technology, Zunyi Normal College, Zunyi 563006, China

**Keywords:** adventitious root, 24-epibrassinolide, reactive oxygen species, polyamine oxidase, respiratory burst oxidase homologues, pepper

## Abstract

Adventitious root (AR) formation is a critical process in cutting propagation of horticultural plants. Brassinosteroids (BRs) have been shown to regulate AR formation in several plant species; however, little is known about their exact effects on pepper AR formation, and the downstream signaling of BRs also remains elusive. In this study, we showed that treatment of 24-Epibrassinolide (EBL, an active BR) at the concentrations of 20–100 nM promoted AR formation in pepper (*Capsicum annuum*). Furthermore, we investigated the roles of apoplastic reactive oxygen species (ROS), including hydrogen peroxide (H_2_O_2_) and superoxide radical (O_2_^•−^), in EBL-promoted AR formation, by using physiological, histochemical, bioinformatic, and biochemical approaches. EBL promoted AR formation by modulating cell-wall-located polyamine oxidase (PAO)-dependent H_2_O_2_ production and respiratory burst oxidase homologue (RBOH)-dependent O_2_^•−^ production, respectively. Screening of *CaPAO* and *CaRBOH* gene families combined with gene expression analysis suggested that EBL-promoted AR formation correlated with the upregulation of *CaPAO1*, *CaRBOH2*, *CaRBOH5*, and *CaRBOH6* in the AR zone. Transient expression analysis confirmed that CaPAO1 was able to produce H_2_O_2_, and CaRBOH2, CaRBOH5, and CaRBOH6 were capable of producing O_2_^•−^. The silencing of *CaPAO1*, *CaRBOH2*, *CaRBOH5*, and *CaRBOH6* in pepper decreased the ROS accumulation and abolished the EBL-induced AR formation. Overall, these results uncover one of the regulatory pathways for BR-regulated AR formation, and extend our knowledge of the functions of BRs and of the BRs-ROS crosstalk in plant development.

## 1. Introduction

Adventitious root (AR) formation is a fundamental process in root biology [1]. Ars are postembryonic roots that form from the non-root organs, and can be found in intact plants during normal development or in response to waterlogging stresses [2,3]. Particularly, Ars are produced in excised plant explants in response to wounding and isolation from the donor plants [4], and thereby have been widely exploited in the form of leaf or stem cuttings to propagate plants [5]. AR formation also occurs indirectly in tissue culture, in which Ars are regenerated from callus [5].

Phytohormones, together with many other internal and external stimuli, coordinate every step of AR formation from the first event of cell reprogramming until emergence and outgrowth [1]. Of these signals, auxin is crucial for many aspects of AR formation, from cell fate acquisition to meristem initiation and emergence [6,7,8]. Like auxin, brassinosteroids (BRs) also function as plant-growth-promoting hormones. BRs are steroid molecules, and play important roles in regulating root system development [9,10,11]. The roles of BRs in primary root and lateral root development have been well documented. BRs could promote primary root growth and lateral root formation at low concentrations but suppress these processes at higher concentrations [12,13]. During root development, there exist strong synergistic interactions between BRs and auxin. BRs interact with auxin by having overlapping activities and sharing target genes [14], or by enhancing auxin transport [12]. Nevertheless, in terms of AR formation, the effect of BRs is not yet well established. In Arabidopsis, application of 24-epibrassinolide (EBL, an active BR), even at very low levels (1 nM and 10 nM), could enhance AR formation [15,16]. In rice, BR biosynthesis is also implicated in the initiation and growth of ARs [17]. Exogenously applied BRs also exhibited positive effects on AR formation in the cuttings of other plant species, including barberry, cucumber, marigold, tomato, basil, and chrysanthemum [9,18,19,20,21]. In contrast, the inhibitory effects of EBL on AR formation were also reported in grapevine cuttings [22]. These studies suggested that BRs indeed participated in AR formation, although the effects of BRs are controversial and should be further determined.

In addition to plant hormones, the regulation of AR formation has also been tightly linked to reactive oxygen species (ROS) [2,23,24]. Hydrogen peroxide (H_2_O_2_) and superoxide radical (O_2_^•−^) are two typical ROS, belonging to non-radical and free radical forms, respectively [25,26]. H_2_O_2_ is the most stable form of ROS, with a lifetime ranging from milliseconds to seconds. O_2_^•−^ is moderately reactive, and its estimated lifetime is of the order of milliseconds [27]. Both H_2_O_2_ and O_2_^•−^ are also the stable forms of ROS, with their lifetimes ranging from milliseconds to seconds [27]. Polyamine oxidase (PAO) catalyzes the catabolism of spermidine and spermine with concomitant production of H_2_O_2_ [28]. PAO-generated H_2_O_2_ has been reported to play important roles in AR formation [29,30]. O_2_^•−^ is produced by plasma-membrane-localized flavin-containing NAPDH oxidases, referred to as respiratory burst oxidase homologues (RBOHs) in plants [27,31]. The roles of RBOH-derived O_2_^•−^ in AR formation have been elucidated in many plant species, such as poplar [32], Arabidopsis [24], apple [33], mung bean [34], and cucumber [23]. ROS-facilitated AR formation is always associated with multiple plant hormones, including auxin, ethylene, salicylic acid, and so on [23,24,34,35,36]. BRs are known to be tightly linked to ROS in other development processes [37]; however, the interactions between BRs and ROS during AR formation have not been reported.

Pepper (*Capsicum annuum* L.) is an economically important vegetable crop, but with poor ability to regenerate [38]. In this work, we investigated the effects of BRs on pepper AR formation, and the roles of cell-wall-PAO-generated H_2_O_2_ and plasma membrane RBOH-generated O_2_^•−^ were further confirmed in this process. Our results of this study provide new insights into the mechanisms underlying the BR-induced AR development in pepper.

## 2. Materials and Methods

### 2.1. Pepper Seedling Growth and Treatment

Pepper (*C. annuum*) cultivar “Guofu 208”, which was obtained from JingyanYinong (Beijing, China) Seed Sci-tech Co. Ltd., was used in this study. Seeds were soaked for 10 h, surface-sterilized with 5% NaClO for 10 min, and washed with distilled water five times, followed by germination on a floating plastic net in darkness. The germinated seeds were sown in vermiculite supplied by ¼-strength Hoagland nutrition solution. The seedlings were then placed in an incubator with photosynthetic active radiation of 200 μmol/m^2^/s, photoperiod of 12/12 h, and temperature of 28 °C/20 °C (day/night) [39]. Four-week-old pepper seedlings were cut at 2–3 mm above the hypocotyl–root junction, with the whole root system and the emerged roots being removed. After washing with distilled water, the pepper explants were placed in a plastic box (20 × 13.5 × 5 cm, L × W × H) containing 800 mL of distilled water, with a submergence depth of 1.5 cm, and then maintained for another ten days. At 10 d post excision of primary root, the number and length of Ars were recorded, and the explants were photographed.

To assess the effect of BRs on pepper AR formation, 24-epibrassinolide (EBL), as one of the most active BRs, was added to the water at concentrations of 5–5000 nM, and Brassinazole (BRz) was applied as a specific inhibitor of BR biosynthesis at concentrations of 1.5 and 5 μM. To assess the involvement of ROS in BR-induced AR formation, potassium iodide (KI, 0.5 mM), N,N’-Dimethylthiourea (DMTU, 2 mM), and catalase (CAT, 200 unit/mg) were applied as ROS scavengers. DPI (diphenyleneiodonium, 10 μM) was used as inhibitor of NADPH oxidase. MDL72527 (100 μM) and 2-HEH (2-hydroxyethylhydrazine, 100 μM) were applied as PAO inhibitors [40,41]. AG (aminoguanidine, 100 μM) was used as the inhibitor of copper amine oxidase (CuAO) [42]. All the above reagents were purchased from Sigma-Aldrich Co. (St. Louis, MO, USA).

### 2.2. Measurement and Histochemical Analysis of H_2_O_2_ and O_2_^•−^

The quantitative measurement of H_2_O_2_ was performed as described previously [43]. O_2_^•−^ content was measured based on the kit instruction (GENMED, Shanghai) using the hydroxylamine oxidation method described previously [25].

H_2_O_2_ and O_2_^•−^ were also detected by histochemical staining with diaminobenzidine (DAB) and nitro-blue tetrazolium (NBT), respectively, according to the published methods [23,43]. After staining, the samples were soaked in 75% ethanol, and photographed under an SZX16 anatomical microscope (Olympus, Tokyo, Japan) equipped with a DP73 digital camera system.

### 2.3. Determination of Cytoplasmic and Cell-Wall PAO Enzymatic Activities

PAO enzymatic activities were determined spectrophotometrically according to the method reported by Yu et al. [44], with some modifications. Briefly, the 5 mm basal hypocotyls were ground with liquid nitrogen and homogenized in 1.5 mL of 0.1 M potassium phosphate buffer (PBS) at pH 6.5. After centrifugation at 12,000× *g* for 10 min at 4 °C, the supernatants were used for measuring extractable cytoplasmic PAO (CP-PAO). The residues were sequentially washed twice by centrifuging in 0.1 M PBS containing 2% (*v/v*) Triton X-100 (Sigma), and then three times in the buffer alone, in order to remove any traces of contaminating extractable cytoplasmic PAO. Cell walls were incubated overnight in 0.1 M PBS containing 1 M NaCl with shaking at 30 °C and centrifuged at 1000× *g*. The supernatant was the ionic cell wall fraction and was used to determine the activities of cell-wall-bound PAO (CW-PAO). The cytoplasmic and CW-PAO activities were measured in a 2 mL reaction system, which contained 0.1 mL of crude enzyme extract, 0.1 mM 4-aminoantipyrine, 1.0 mM 3,5-dichloro-2-hydroxybenzenesulphonic acid (DCHBS), and 10 mg horseradish peroxide (≥250 units/mg; Sigma-Aldrich). The reactions were initiated by adding 200 mM spermidine. Protein concentrations were determined with a Protein Assay Kit II (Bio-Rad, Hercules, CA, USA). One unit of PAO activity (U) was defined as a change in absorbance of 0.01 optical density.

### 2.4. Assay of Plasma Membrane (PM) NADPH Oxidase Activity

The protein was isolated and determined according to the procedure for the Plant Plasma Membrane Protein Extraction Kit (BestBio, Shanghai, China). PM-NADPH oxidase activity was determined following the superoxide dismutase (SOD)-inhibitable and NADPH-dependent oxidation of {sodium, 3′-[1-(phenylamino-carbonyl)-3,4-tetrazolium]-bis(4-methoxy-6-nitro) benzene-sulphonic acid hydrate} (XTT) by O_2_^•−^, as described previously [45].

### 2.5. Genome-Wide Identification of CaPAO and CaRBOH Genes in Pepper

The Arabidopsis PAO (AtPAO) proteins were obtained from TAIR database (https://www.arabidopsis.org/, accessed on 15 March 2023), and the tomato PAO (SlPAO) proteins were obtained from Sol Genomics Network (SL4.0; https://solgenomics.net/organism/Solanum_lycopersicum/genome/, accessed on 15 March 2023). All AtPAO and SlPAO sequences were used as baits for searching CaPAO homologs in the *Capsicum annuum* genome (assembly Pepper Zunla 1, V1.0) in NCBI (https://www.ncbi.nlm.nih.gov/genome/10896, accessed on 15 March 2023). A multiple-sequence alignment was performed using ClustalX (version 1.81) [46]. A phylogenetic tree was constructed using MEGA (version 7.0) to perform the neighbor-joining (NJ) method with 100 bootstrapped replicates [47]. Gene information for phylogenetic analysis is listed in Supplementary Table S1. Genome-wide identification of *CaRBOH* genes was performed by using the same strategy as *CaPAO*s. The secretory signal peptides of AtPAO1, SlPAO1, and CaPAO1 proteins were predicted via SignalP-5.0 (https://services.healthtech.dtu.dk/service.php?SignalP-5.0, accessed on 15 March 2023), and the transmembrane domains of CaRBOH proteins were predicted by TMHMM-2.0 program (https://services.healthtech.dtu.dk/services/TMHMM-2.0, accessed on 15 March 2023).

### 2.6. qRT-PCR Analysis

Total RNA was isolated from 5 mm segments from the base of pepper hypocotyls at the indicated times during adventitious root formation. Total RNA was isolated using an EasyPure Plant RNA Kit (Transgen, Beijing, China) according to the manufacturer’s protocol. Residual genomic DNA was digested with Dnase I (Sigma-Aldrich). Reverse transcription was performed using 2 mg of total RNA and M-MLV Reverse Transcription System (Promega, Madison, WI, USA). Quantitative PCR (qPCR) was performed using the TransStart Green qPCR SuperMix (Transgen) on a LightCycler 96 machine (Roche Diagnostics, Basel, Switzerland), according to the manufacturer’s instructions. Three biological replicates were performed per gene, and three technical replicates were performed within an experiment. Samples were collected from three independent experiments. The relative transcript abundance was calculated based on 2^−∆∆CT^ threshold cycle method. The *CaUBI-3* gene (LOC107873556) was used as an internal control [39,48]. The expression level of each gene under specific treatment was presented as a value relative to the control. The primer information is listed in Supplementary Table S2.

### 2.7. Transient Expression of CaPAO1 and CaRBOHs in Pepper Leaves

The full-length cDNAs of *CaPAO1*, *CaRBOH2*, *CaRBOH5*, and *CaRBOH8* genes were cloned into pCAMBIA2300 vector. The specific primers are listed in Supplementary Table S3. Then, the recombined vector was transformed into *Agrobacterium tumefaciens* strain GV3101, which was further infiltrated into the 1st true leaves of 20-day-old pepper seedlings by a needleless syringe. After 3 d of infiltration, the leaves were harvested for RNA extraction, and the expression levels of target genes were detected by qRT-PCR. The leaves were also used for histochemical staining of H_2_O_2_ and O_2_^•−^.

### 2.8. Virus-Induced Gene Silencing (VIGS) of CaPAO1 and CaRBOHs in Pepper

With the full-length cDNA of target gene as template, a 300 bp fragment was amplified using the specific primers listed in Supplementary Table S3. The site was chosen by SGN VIGS Tool software (https://vigs.solgenomics.net/, accessed on 5 May 2023). PCR products were further cloned into TRV2 vector with restriction enzymes *EcoR* I and *BamH* I, resulting in the pTRV2 derivatives pTRV2-*CaPAO1*, pTRV2-*CaRBOH2*, pTRV2-*CaRBOH5*, and pTRV2-*CaRBOH6*. The transformed Agrobacterial cells (GV3101) were collected and resuspended in infiltration buffer (10 mM MES, pH 5.6, 10 mM MgCl_2_, 200 μM acetosyringone) to a final density of OD_600_ = 1.0, and then left at room temperature for 3 to 4 h without shaking. Before infiltration, *A. tumefaciens* cultures containing pTRV1 and pTRV2 or their derivatives were mixed in a 1:1 ratio. The culture suspensions were infiltrated into the germinated seeds (with 0.5~1 cm of radicles) using a vacuum-assisted infiltration method [49]. After completion of agro-inoculation, the seeds were sown in the 32-well trays containing peatlite mixes (60% peatmoss, 20% vermiculite, and 20% perlite), and grown at 22 °C under a 16 h/8 h light/dark cycle in a controlled environmental chamber. After three weeks of cultivation, the VIGS plants were cut above the cotyledon node, and the resulting explants were used to assay the AR formation capacity of epicotyls. Before AR formation assay, qRT-PCR was performed to ensure silencing efficiency.

### 2.9. Statistical Analysis

Each datum is shown as mean ± standard deviation (SD) for at least three replicates. Two-way analysis of variance was performed in GraphPad Prism or with SPSS statistics (SPSS 19.0). Student’s *t*-tests were performed in Microsoft Excel. In graphs that include letters, each letter represents a statistically significant mean by Tukey’s test at a level of *p* < 0.05.

## 3. Results

### 3.1. EBL Promoted AR Formation in Pepper

**Figure 1:**
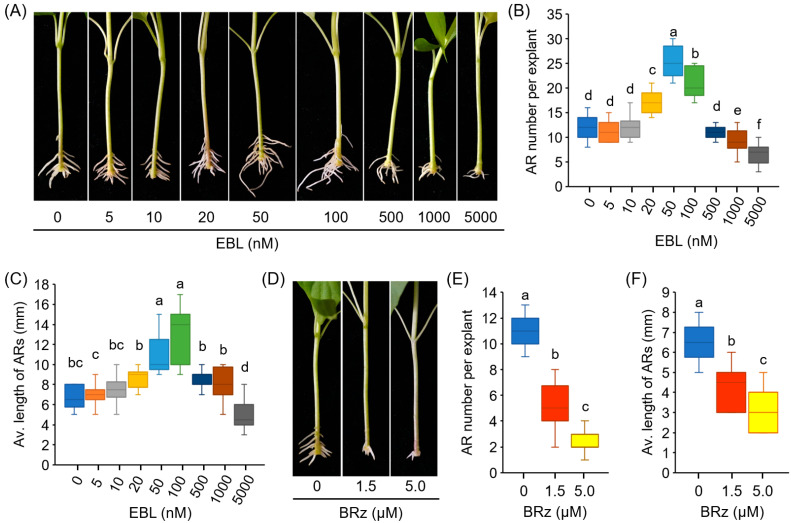
EBL promotes AR formation in the hypocotyls of pepper explants. (**A**–**C**) Effects of EBL on AR formation of pepper. Pepper explants with primary root excision were treated with EBL at different concentrations (0–5000 nM) for 10 days, followed by photographing (**A**), counting of AR number (**B**), and determination of AR average length (**C**). (**D**–**F**) Effects of BRz on AR formation of pepper. Pepper explants were treated with BRz at concentrations of 1.5 and 5.0 μM for 10 days. Each treatment has 15 pepper explants. Different lowercase letters indicate that the values were significantly different among different treatments (*p* < 0.05).

EBL induced significant increases in AR number and AR length in a dose-dependent manner. EBL at 50 nM exhibited the greatest effect (Figure 1A–C). Treatment with EBL at concentrations lower than 10 nM led to no significant difference in the AR number and length, while the pepper explants treated with relatively high concentrations of EBL (1 and 5 μM) developed less and shorter Ars (Figure 1A–C).

BRz, a specific inhibitor of BR biosynthesis, was also used to determine the effect of BR on AR formation. As shown in Figure 1D–F, a remarkable repression of AR number and AR length was observed after BRz treatment. These results suggested that exogenous EBL treatment promoted AR formation in pepper explants.

### 3.2. ROS was Involved in EBL-Promoted AR Formation

**Figure 2:**
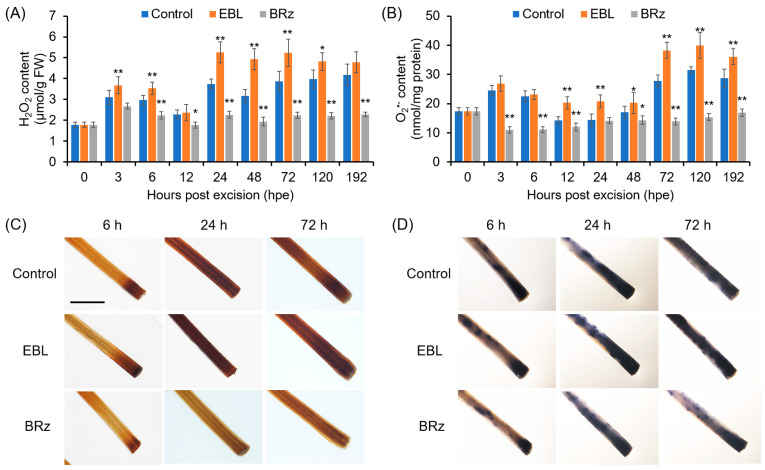
Kinetics of changes in H_2_O_2_ and O_2_^•−^ contents during AR formation in EBL- or BRz-treated pepper explants. (**A**,**B**) Contents of H_2_O_2_ (**A**) and O_2_^•−^ (**B**) in the AR zone of pepper hypocotyls after different durations of EBL or BRz treatments. (**C**,**D**) Histochemical staining of H_2_O_2_ (**C**) and O_2_^•−^ (**D**) in the AR zone after different durations of EBL or BRz treatments. Once primary roots were cut, pepper explants were treated with water (Control), EBL (50 nM), or BRz (5 μM), and used for AR formation. Hypocotyl samples were harvested at indicated hours (h) after treatment. One asterisk (*) and two asterisks (**) in (**A**,**B**) indicate that the mean values of three replicates were significantly different between control and treatment at each time point at *p* < 0.05 and *p* < 0.01, respectively. FW, fresh weight. The scale bar in (**C**,**D**) is 5 mm.

During AR formation, H_2_O_2_ contents in the rooting zone increased slightly at 3 h and 6 h, and then, at 24 h, the rate of H_2_O_2_ production increased sharply. In the EBL-treated hypocotyls, the amounts of H_2_O_2_ were much more prevalent than in the control explants, while in the BRz-treated explants, the H_2_O_2_ accumulation after 24 h was significantly repressed (Figure 2A). For O_2_^•−^, the accumulation pattern in the control explants was similar to H_2_O_2_, but EBL treatment enhanced O_2_^•−^ accumulation after 72 h in the rooting zone (Figure 2B). Again, BRz was effective in repressing O_2_^•−^ accumulation (Figure 2B). EBL-induced and BRz-repressed accumulation of H_2_O_2_ and O_2_^•−^, detected in situ by DAB and NBT staining, respectively, were also observed clearly in the AR zone (Figure 2C,D).

**Figure 3:**
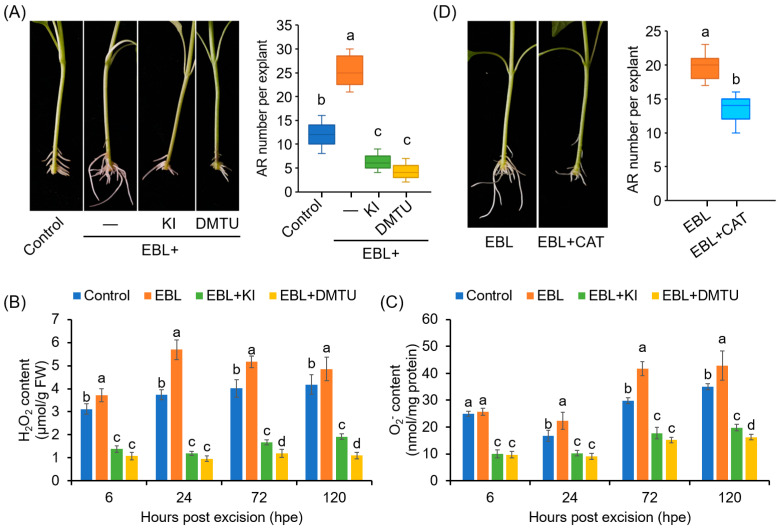
Involvement of ROS in EBL-promoted AR formation in pepper explants. (**A**) Effects of KI and DMTU on EBL-induced AR formation. Once primary roots were cut, pepper explants were used for AR formation in water without (Control) or with EBL (50 nM), EBL + KI (0.5 mM), or EBL + DMTU (2 mM) for 10 days, followed by photographing phenotype and counting AR number. KI and DMTU were used as ROS scavengers. KI, potassium iodide; DMTU, *N*,*N*’-Dimethylthiourea. (**B**,**C**) Contents of H_2_O_2_ (**B**) and O_2_^•−^ (**C**) in the AR zone of pepper explants treated with EBL, EBL + KI, or EBL + DMTU for 6, 24, 48, and 120 h. (**D**) Effects of CAT on EBL-induced AR formation. AR formation in pepper explants treated with EBL (50 nM) or EBL + CAT (200 unit/mg) for 10 days, followed by photographing phenotype and counting AR number. Each treatment has 15 pepper explants. Different lowercase letters indicated that the values were significantly different among different treatments (*p* < 0.05).

To determine whether ROS accumulation contributes to EBL-induced AR formation in pepper hypocotyls, we analyzed the effects of KI and DMTU on AR formation. Co-treatment with KI or DMTU completely abolished the promoting effects of EBL on AR number (Figure 3A), and compromised the EBL-induced accumulation of H_2_O_2_ and O_2_^•−^ in the rooting zone (Figure 3B,C). These results suggested that ROS, including H_2_O_2_ and O_2_^•−^, contributed to EBL-promoted AR formation in pepper. It should also be noted that treatment with KI or DMTU alone inhibited AR formation in pepper (Supplementary Figure S1).

Furthermore, EBL-induced AR formation was also markedly inhibited by CAT, another ROS scavenger (Figure 3D). Considering that CAT functions extracellularly [50], our results suggested that the ROS generated in the cell wall and plasma membrane were associated with the EBL-induced AR formation in pepper.

### 3.3. CW-PAO and PM-NADPH Oxidase Were Involved in EBL-Induced ROS Generation during AR Formation

**Figure 4:**
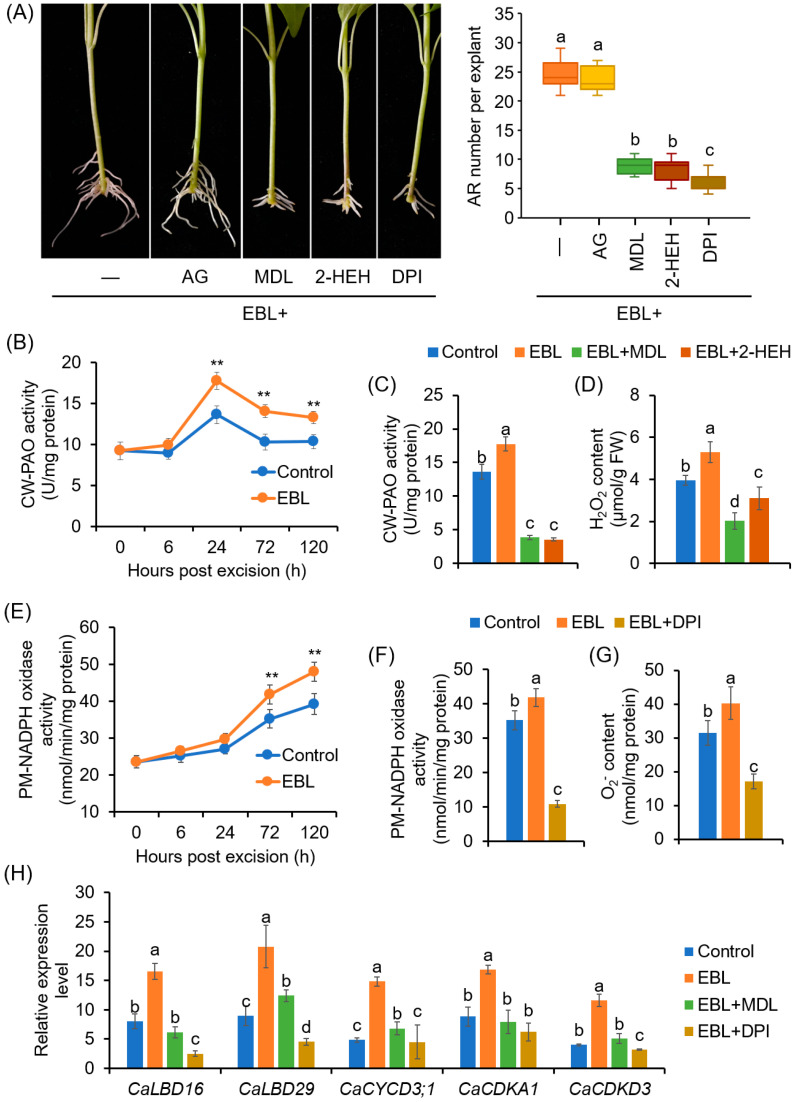
Involvement of PAO-dependent H_2_O_2_ and NADPH oxidase-dependent O_2_^•−^ in EBL-promoted AR formation in pepper explants: (**A**) Effects of AG, MDL72527, 2-HEH, and DPI on EBL-induced AR formation. Once primary roots were cut, pepper explants were used for AR formation in water with EBL (50 nM), EBL + AG (100 μM), EBL + MDL72527 (MDL, 100 μM), EBL + 2-HEH (100 μM), or EBL + DPI (10 μM) for 10 days, followed by photographing phenotype and counting AR number. DPI and AG were inhibitors of copper amine oxidase and NADPH oxidase, respectively; MDL and 2-HEH were PAO inhibitors. AG, aminoguanidine; 2-HEH, 2-hydroxyethylhydrazine; DPI, diphenyleneiodonium. (**B**) Effects of EBL on the activities of cell-wall PAO (CW-PAO) during AR formation. (**C**,**D**) Effects of MDL72527 and 2-HEH on EBL-induced CW-PAO activity (**C**) and H_2_O_2_ content (**D**) at 24 h of AR formation. (**E**) Effects of EBL on the activities of plasma membrane NADPH oxidase (PM-NADPH oxidase) during AR formation. (**F**,**G**) Effects of DPI on EBL-induced PM-NADPH oxidase activity (**F**) and O_2_^•−^ content (**G**) at 72 h of AR formation. (**H**) Expression of *CaLBD*, *CaCYCLIN*, and *CaCDK* genes in response to EBL, EBL + MDL, and EBL + DPI treatments at 72 h of AR formation. The expression level for each gene in the mock plants at 0 dpe was normalized to 1.0. The accession numbers for these genes are listed in Table S4. Each treatment contains three biological replicates, and each replicate has 15 explants. Different lowercase letters in (**A**,**C**,**D**,**F**–**H**) indicate that the mean values of three replicates are significantly different among different treatments (*p* < 0.05). Two asterisks (**) in (**B**,**E**) indicate significant differences between control and EBL treatment at *p* < 0.01.

To investigate which enzymatic pathway was involved in EBL-induced ROS production, the effects of AG (an inhibitor of CuAO), MDL 72,527 and 2-HEH (inhibitors of PAO), and DPI (an NADPH oxidase inhibitor) on EBL-induced AR formation were evaluated. The results suggested that MDL72527, 2-HEH, and DPI, but not AG, could significantly compromise the promoting effect of EBL on AR formation (Figure 4A), indicating that EBL-induced AR formation involved the production of ROS mainly mediated by PAOs and NADPH oxidases but not by CuAOs. Compared to the control, treatment with MDL72527 or DPI alone also prohibited AR formation (Supplementary Figure S1).

Next, the changes in the enzymatic activities of PAO and NADPH oxidase in the rooting zone in response to EBL treatment were detected. The activity of CW-PAO increased significantly after 24 h, and EBL significantly enhanced CW-PAO activity (Figure 4B). At 24 h of AR formation, both MDL72527 and 2-HEH application could repress the activity of CW-PAO (Figure 4C) and H_2_O_2_ content (Figure 4D). However, for CP-PAO, during AR formation, its activities increased after 6 h, and peaked at 24 h, but EBL exhibited no significant effect on CP-PAO activity (Supplementary Figure S2).

For PM-NADPH oxidase, the activities also began to increase at 24 h, but a sharp increase occurred at 72 h. Again, EBL treatment could enhance NADPH oxidase activity (Figure 4E). DPI application significantly inhibited PM-NADPH oxidase activities and O_2_^•−^ generation at 72 h of AR formation (Figure 4F,G). Moreover, EBL upregulated the expression of genes involved in AR primordia cell-fate decision and cell division, including *CaLBD16*, *CaLBD29*, *CaCYCD3;1*, *CaCDKA1*, and *CaCDKD3* (Figure 4H). And MDL72527 and DPI could repress the promoting effects of EBL on the expression levels of these genes (Figure 4H). These results suggested that CW-PAO-generated H_2_O_2_ and PM-NADPH oxidase-generated O_2_^•−^ were associated with EBL-induced AR formation in pepper explants.

### 3.4. Identification of EBL-Targeted CaPAO during Pepper AR Formation

**Figure 5:**
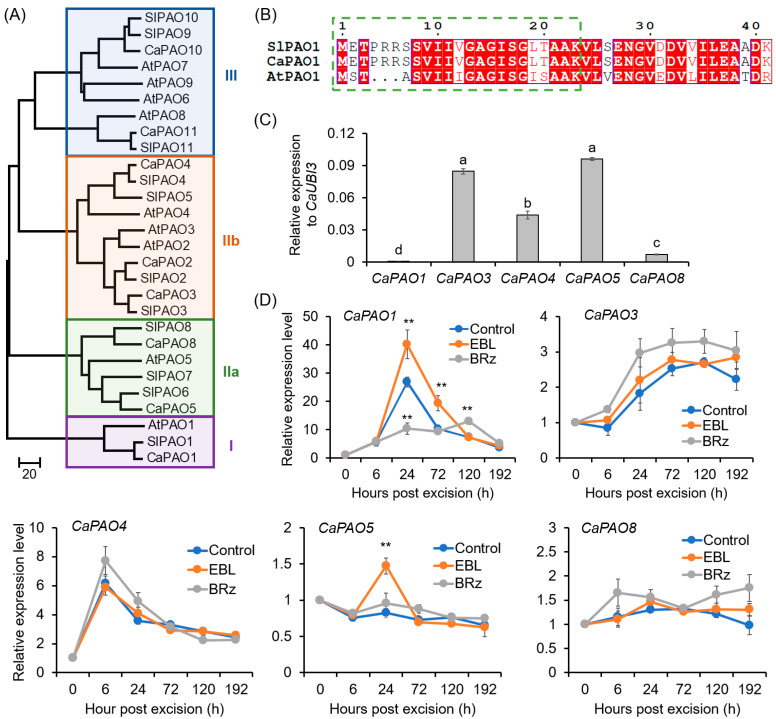
Phylogenetic analysis of CaPAO family and EBL-induced expression of *CaPAO*s during AR formation in pepper explants: (**A**) Neighbor-joining phylogenetic tree of the CaPAO, AtPAO, and SlPAO proteins. The accession numbers for these proteins are listed in Table S1. (**B**) The secretory signal peptide in the N-terminal of CaPAO1 and SlPAO1 proteins. The green dashed square indicates the secretory signal peptide. Invariant residues are shaded in blue boxes, with residues that are conserved colored red, and variable residues shown in black. (**C**) Expression levels of *CaPAO* genes in the base of pepper hypocotyls before AR formation. The expression level of each *CaPAO* gene was normalized to *CaUBI3* expression. (**D**) Effects of EBL and BRz on the expression of *CaPAO* genes during AR formation. The expression level at 0 h was normalized to 1.0. Each treatment contains three biological replicates, and each replicate has 15 explants. Different lowercase letters in (**C**) indicate that the mean values of three replicates are significantly different among different genes (*p* < 0.05). Two asterisks (**) in (**D**) indicate significant differences between control and EBL or BRz treatment at *p* < 0.01.

A total of eight CaPAO candidates with typical amnio acid domains were obtained from the pepper genome (Figure 5A; Supplementary Figure S3). CaPAO10 and CaPAO11, which contained a SWIRM domain and belonged to the subgroup III, were characterized as histone lysine-specific demethylase (Figure 5A; Supplementary Figure S3) [25,51], and thus not included in our next study. The remaining six CaPAOs (CaPAO1–5 and CaPAO8) were characterized as typical PAOs (Supplementary Figure S3), and they were distributed in three subgroups (I, IIa, and IIb; Figure 5A). Of them, CaPAO1 was clustered into subgroup I, with SlPAO1 and AtPAO1. For SlPAO1 and CaPAO1, a secretory signal peptide was predicted at their N-terminals (Figure 5B). For SlPAO1, its apoplastic localization had also been identified by Chen et al. [25].

For the *CaPAO* genes, it was detected that *CaPAO1*, *CaPAO3*, *CaPAO4*, *CaPAO5*, and *CaPAO8* were expressed in the basal region of hypocotyls in pepper seedlings (Figure 5C). During AR formation, the expression of *CaPAO1* increased 28.8-fold at 24 h and then decreased. EBL exerted a significant enhancing effect on the expression of *CaPAO1*, while in the BRz-applied explants, the expression peak of *CaPAO1* at 24 h was almost abolished (Figure 5D). For *CaPAO3* and *CaPAO4*, although their expression levels increased during AR formation, EBL treatment could not exert a significant effect on their expression (Figure 5D). For *CaPAO5* and *CaPAO8*, their expression did not change significantly (Figure 5D). Combining these results, it was suggested that *CaPAO1* might be involved in EBL-induced AR formation.

### 3.5. Identification of EBL-Targeted CaRBOHs during Pepper AR Formation

**Figure 6:**
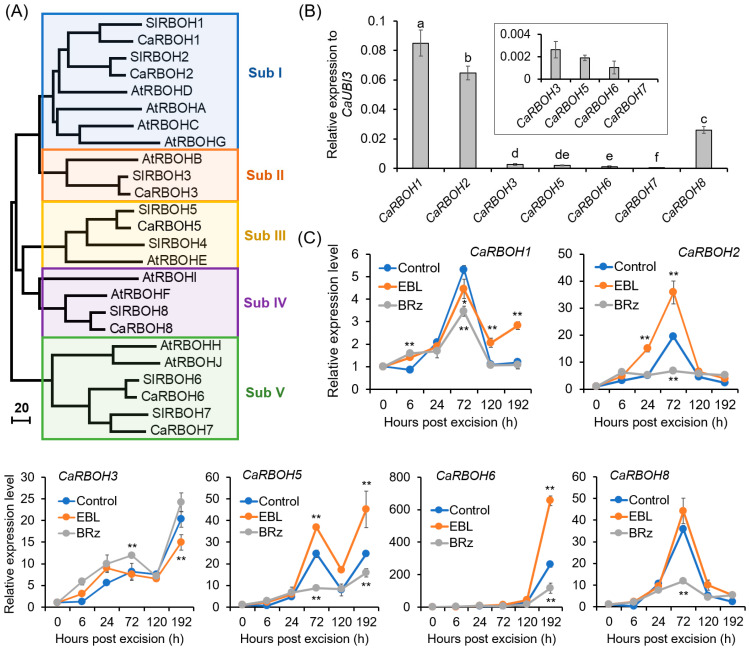
Phylogenetic analysis of CaRBOH family and EBL-induced expression of *CaRBOH*s during AR formation in pepper explants. (**A**) Neighbor-joining phylogenetic tree of CaRBOH, AtRBOH, and SlRBOH proteins. The accession numbers of these proteins are listed in Supplementary Table S1. (**B**) Expression levels of *CaRBOH* genes in the base of pepper hypocotyls before AR formation. The expression level of each gene was normalized to *CaUBI3* expression. (**C**) Effects of EBL and BRz on the expression of *CaRBOH* genes during AR formation in pepper hypocotyls. The expression level for each gene at 0 h was normalized to 1.0. Each treatment contains three biological replicates, and each replicate has 15 explants. Different lowercase letters in (**B**) indicate that the mean values of three replicates are significantly different among different genes (*p* < 0.05). One asterisk (*) and two asterisks (**) in (**C**) indicate significant differences between control and EBL or BRz treatment at *p* < 0.05 and *p* < 0.01, respectively.

A total of seven *CaRBOH* genes were retrieved from the pepper genome (Figure 6A), consistent with a previous report [52]. CaRBOHs were clustered into five subfamilies (I–V), based on phylogenetic analysis (Figure 6A) and motif distribution (Supplementary Figure S4). All the CaRBOHs were transmembrane proteins with typical conserved domains (Supplementary Figure S5). In the basal region of the hypocotyls of pepper seedlings, all the *CaRBOH* genes were expressed, and of them, *CaRBOH1*, *CaRBOH2*, and *CaRBOH8* were expressed at a relatively high level, but for *CaRBOH7*, its expression was very low (Figure 6B).

The temporal changes in the expression levels of *CaRBOH*s during AR formation and their expression patterns in response to EBL and BRz treatments are shown in Figure 6C. The expression of *CaRBOH1*, *CaRBOH2*, and *CaRBOH8* started to increase at 24 h, peaked at 72 h, and then declined. Of them, *CaRBOH2* showed a 1.84-fold increase after EBL treatment at 72 h, but significantly decreased in BRz-treated explants compared with the control. For *CaRBOH3* and *CaRBOH6*, their expression increased gradually, and at 192 h, a 20.31- and 262.46-fold increase was detected, respectively. EBL could induce and BRz could repress the expression of *CaRBOH6*. For *CaRBOH5*, its expression showed a substantial increase at both 72 and 192 h, and a significant induction was observed after EBL treatment. Combining these results, these results suggest that *CaRBOH2* might be involved in EBL-induced AR initiation, *CaRBOH6* might be involved in EBL-induced AR elongation, and *CaRBOH5* might be involved in EBL-induced AR initiation and elongation.

### 3.6. Capacities of CaPAO1 and CaRBOHs in ROS Production in Pepper Leaves

**Figure 7:**
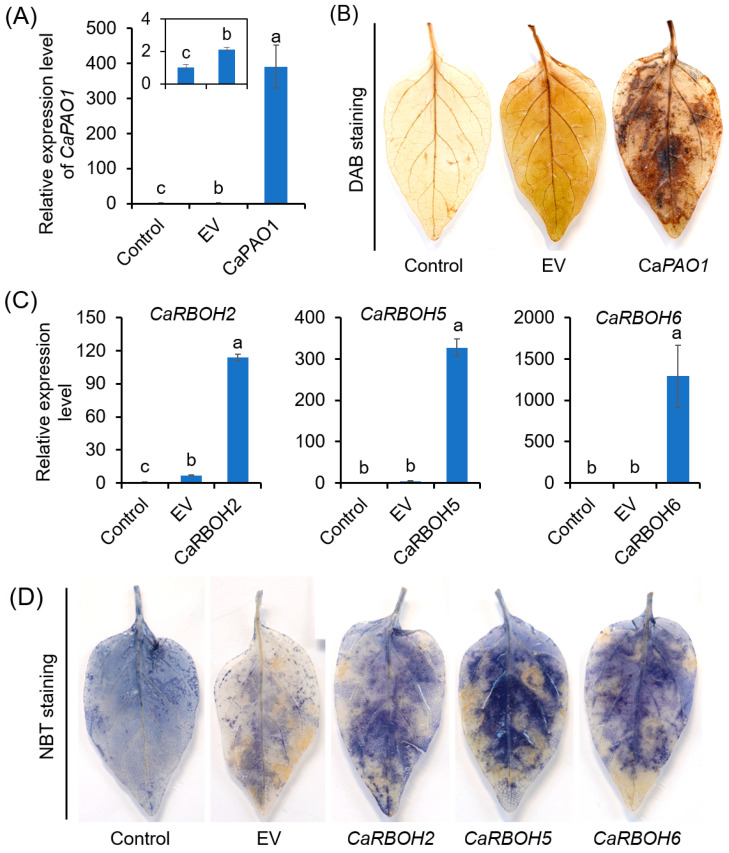
Capability of CaPAO1, CaRBOH2, CaRBOH5, and CaRBOH6 in ROS production based on transient expression analysis. (**A**,**B**) Relative expression level of *CaPAO1* (**A**) and detection of endogenous H_2_O_2_ by DAB staining (**B**) in pepper leaves expressing *CaPAO1*. (**C**,**D**) Relative expression levels of *CaRBOH2*, *CaRBOH5*, and *CaRBOH6* (**C**) and detection of endogenous O_2_^•−^ by NBT staining (**D**) in pepper leaves expressing *CaRBOH2*, *CaRBOH5*, and *CaRBOH6*. Control indicates the leaves without infiltration, and EV indicates infiltration of leaves with *Agrobacterium* carrying the empty vector. Each treatment has 15 pepper explants. In (**B**,**D**), the results show similar trends, and a representative result is shown. Different lowercase letters in (**A**,**C**) indicate that the mean values of three replicates were significantly different among different treatments (*p* < 0.05).

The capabilities of *CaPAO1*, *CaRBOH2*, *CaRBOH5*, and *CaRBOH6* in ROS production were evaluated using transient expression analysis (Figure 7). DAB staining suggested that pepper leaves expressing *CaPAO1* showed a higher H_2_O_2_ level than the control and empty vector control (EV) (Figure 7A,B). Likewise, we found that pepper leaves with expression of *CaRBOH2*, *CaRBOH5*, and *CaRBOH6* showed a remarkable increase in O_2_^•−^, detected with NBT staining (Figure 7C,D). These results confirm the ability of CaPAO1 in H_2_O_2_ generation, and the capacity of CaRBOH2, CaRBOH5, and CaRBOH6 in O_2_^•−^ production.

### 3.7. Identification of the Capabilities of CaPAO1 and CaRBOHs in EBL-Induced AR Formation in Pepper

We employed VIGS to investigate the function of the *CaPAO1* and *CaRBOH* genes in EBL-induced AR formation. In a preliminary experiment, we silenced the expression of the phytoene desaturase (*PDS*) gene, which is used as a visible marker to monitor VIGS efficiency [53]. As shown in Supplementary Figure S6A, the *TRV2:PDS* plants exhibited a significant etiolation phenotype above the cotyledon code. These results indicated that the VIGS system worked efficiently under our experimental conditions, and gene silencing occurred in the epicotyls but not hypocotyls. Thereby, in our following studies, the epicotyls were used in the AR formation assay.

**Figure 8:**
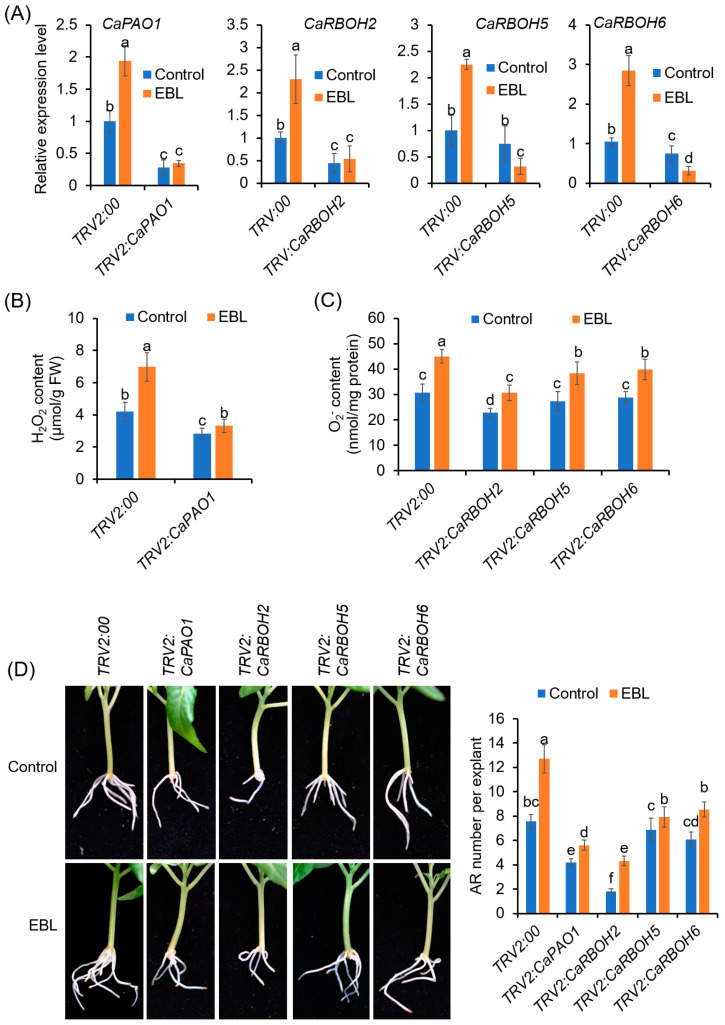
Silencing of *CaPAO1* and *CaRBOH*s decreases AR formation in pepper. (**A**) Expression levels of *CaPAO1* and *CaRBOH*s in the epicotyls of control and VIGS plants. (**B**) H_2_O_2_ contents in the AR zone of *CaPAO1*-silenced explants at 24 h of AR formation. (**C**) O_2_^•−^ contents in the AR zone of control and *CaRBOH*-silenced explants at 72 h of AR formation. (**D**) AR formation in the control and VIGS explants treated with or without EBL. Each treatment has 15 pepper explants. Different lowercase letters indicate that the mean values of ten replicates are significantly different among different treatments (*p* < 0.05).

Thus, we used this VIGS system to inhibit the expression of the *CaPAO1* and *CaRBOH* genes in pepper. Under normal conditions, the growth of VIGS plants was comparable to that of the control plants (Supplementary Figure S6B). In the *TRV2:CaPAO1* explants, compared to the *TRV2:00* explants, the expression of *CaPAO1* at 24 h of AR formation was significantly decreased, both in the explants without and with EBL treatments, confirming that the *CaPAO1* gene was successfully silenced (Figure 8A). The similarly reduced expression of target genes was also detected in the *TRV2:CaRBOH* explants (Figure 8A). In accordance with the decreased gene expression, the H_2_O_2_ contents at 24 h and O_2_^•−^ contents at 72 h were significantly reduced in the AR zone (Figure 8B).

Compared with the *TRV2:00* control explants, AR formation was significantly repressed in the *TRV2: CaPAO1* and *TRV2: CaRBOH2* explants, both with and without EBL application (Figure 8C,D). For the *TRV2:CaRBOH5* and *TRV2:CaRBOH6* explants, although the AR formation capacity was not significantly reduced without EBL treatment, the promoting effects of EBL on AR formation were significantly abrogated (Figure 8C,D). These results confirmed the function of *CaPAO1*, *CaRBOH2*, *CaRBOH5*, and *CaRBOH6* in EBL-induced AR formation in pepper.

## 4. Discussion

AR formation is a critical developmental process in cutting propagation within the horticultural industry [5]. Our study reveals an important role of BRs in promoting AR formation in pepper explants. We further show that cell-wall-PAO-derived H_2_O_2_ and plasma-membrane-RBOH-derived O_2_^•−^ functioned as the second messengers that regulate gene expression and participate in adventitious root initiation and elongation.

### 4.1. BRs Promoted AR Formation in a Dose-Dependent Manner

The data reported in this study demonstrate the promoting effects of EBL on AR formation in pepper explants. Exogenous EBL enhanced the number and length of AR at low concentrations (≤100 nM), while high concentrations (≥1 μM) of EBL treatments suppressed AR development (Figure 1). These results suggested that the effects of EBL on AR formation were dose-dependent. Consistent with our results, Guan and Roddick reported that application of a low concentration of EBL (≤0.1 μM) increased the AR number and length in tomato, while application of EBL in excess of 1 μM reduced root growth as well as root number and root length [54]. The effects of other forms of BRs on AR formation were also reported in other plant species. For example, in Norway spruce trees, treatment with (22S,23S)-28-homobrassinolide (SSHB), another synthetic form of BRs, significantly enhanced AR formation at concentrations of 3–60 mg/L (approximately 6–120 µM) [55]. In cucumber, exogenous application of 1 μM BR significantly promoted AR formation, while high concentrations of BR (2–8 μM) effectively inhibited it [21]. In marigold, EBL significantly increased the AR number at 0.1, 0.5, and 1 μM, but exerted no significant effect at 5 μM [20]. Combining the previous and present research data, the results indicate that BRs have controversial roles, depending on the BR concentrations, plant species, plant growth conditions, and developmental stages [56]. In plants, the endogenous BR contents varied in a range of 0.05 ng/g FW to 0.6 nmol/kg FW (approximately 0.3 ng/g FW) [57,58]. It should be noted that a µM concentration is far above the physiological levels and is documented to be toxic. Nevertheless, the optimum concentrations of BRs for AR formation were quite different in these studies. This might be due to the sensitivity of different plant species to BRs, as well as the different BR forms applied.

Moreover, BRs have been shown to regulate AR formation through crosstalk with other plant hormones. Auxin is the key hormone that induces AR formation [7]. BRs and auxin worked synergistically in many aspects of root development [14]. In Arabidopsis, exogenous EBL could stimulate AR formation in the IAA-overproducing *gulliver1/sur2–7* mutants, likely by enhancing IAA biosynthesis [16]. Treatment with exogenous BRs could restore the expression of auxin-responsive genes involved in root development [59]. Also, the modulation of polar auxin transport is another mechanism by which BRs regulate root development [60]. In addition to auxin, nitric oxide (NO) plays very important roles in BR-induced AR formation. In cucumber, EBL treatment could enhance endogenous NO production. Co-treatments with EBL and the NO donor enhanced AR formation, while the NO scavenger and inhibitors inhibited the positive effects of EBL on adventitious rooting [21]. However, there are also some contradictory results. In Arabidopsis, the promoting effects of EBL on AR formation under cadmium stress were not further enhanced by the NO donor, indicating that there was no synergistic effect between BR and NO [15]. During AR formation in pepper, the interactions between BRs and other phytohormones are less revelatory, so we will largely focus on interpreting the interactions.

In addition to exogenous BRs, the endogenous BR signal was also involved in AR formation in pepper, based on our finding that BRz, an BR inhibitor, could significantly reduce the AR number (Figure 1). Accordingly, it has been reported that BR biosynthesis was implicated in the initiation and growth of ARs in rice [17], although further quantitative detection of BR contents and the expression of corresponding genes involved in BR biosynthesis and receptor is still needed.

### 4.2. BRs Promoted AR Formation through Apoplastic CaPAO1- and CaRBOHs-Derived ROS

ROS are versatile signaling molecules in plants and have been considered as triggers for AR formation, as reviewed recently [61,62]. BRs have been reported to regulate plant stress response and embryonic root development via regulating ROS homeostasis [45,63,64]. However, the cross-regulation between BRs and ROS in AR formation has received little attention. In this study, we found that ROS, including H_2_O_2_ and O_2_^•−^, were required for EBL-induced AR formation in pepper (Figure 2), in agreement with previous reports in cucumber and mung bean [23,65]. EBL enhanced the ROS level while the suppression of ROS counteracted the promoting effect of EBL on AR formation (Figure 2 and Figure 3). Furthermore, we found that apoplastic ROS signals were essential for EBL-induced AR formation, based on the finding that exogenous application with CAT could abolish the promoting effect of EBL on AR formation (Figure 3D). Thus, we revealed that EBL promoted AR formation through the apoplastic ROS-dependent pathway in pepper seedlings.

Apoplastic ROS are engaged in plant development and responses to extrinsic signals. The low antioxidant efficiency in the apoplast allows ROS to accumulate easily [66]. The apoplastic ROS can be channeled into the cytoplasm by multiple PIP-type aquaporins. And H_2_O_2_, which is a neutral molecule, can also diffuse through the plasma membrane to the cell [67,68]. Once they enter the cells, ROS could boost the intracellular ROS pool and activate symplastic ROS signaling [66]. During plant root development, ROS have been reported to be involved in multiple stages, including cell proliferation, elongation, and differentiation, through influencing the gene expression of *CYC*s and *CDK*s and governing the interphasic transition in the cell cycle [69]. AR initiation started from the founder cell specification followed by cell division [70], and *LBD* gene expression leads to AR initiation via the promotion of cell division and the establishment of root primordium identity [71]. In this study, EBL induced the expression of *LBD* genes and cell-cycle-related genes (*CaCYCs*, *CaCDK*s) in a ROS-dependent manner (Figure 4H). ROS might regulate the expression of the *CYC* and *CDK* genes directly via the TEOSINTE BRANCHED1-CYCLOIDEA-PROLIFERATING CELL FACTOR1 (TCP) transcription factor. When ROS levels increased, a disulfide bond was formed, preventing the binding of TCPs on the target gene promoter [69,72]. Taken together, we concluded that EBL triggers ROS accumulation in the rooting region, which further enables the activation of cell specification and division to start AR formation.

In this study, we found that two enzymatic pathways contribute to apoplastic ROS production. One is the cell-wall-located CaPAO1, which metabolizes polyamines with H_2_O_2_ as byproduct. EBL induced the most significant increase in CW-PAO activity and *CaPAO1* expression in the AR zone (Figure 4 and Figure 5). Transient expression significantly confirmed the capability of CaPAO1 in H_2_O_2_ production (Figure 7), and both the pharmacological study with PAO inhibitors and the VIGS analysis indicated the roles of CaPAO1-produced H_2_O_2_ in EBL-induced AR formation (Figure 4 and Figure 8). In the pepper genome, eight *CaPAO* genes were identified, and of them, only CaPAO1 was grouped in subfamily I, together with SlPAO1 from tomato and AtPAO1 from Arabidopsis (Figure 5). SlPAO1 with predicated apoplastic localization was supposed to catalyze the terminal catabolism of polyamines [25]. Accordingly, in the terminal of CaPAO1 and SlPAO1, a highly conserved secretory signal peptide was found (Figure 5). And although AtPAO1 has a predicted cytosolic localization [28], both CaPAO1 and AtPAO1 have a similar gene organization to that of the extracellular ZmPAO and OsPAO [73]. For AtPAO1, it could oxidize Spm but not Spd, and preferred two “uncommon” polyamines (norspermine and thermospermine) as its substrate [74]. These uncommon polyamines are involved in important plant developmental processes, including cell wall patterning, cell death, and xylem cell morphology, as well as disease resistance [75,76]. Considering the high sequence similarity between CaPAO1 and AtPAO1, we could conclude that CaPAO1 may have potential to produce H_2_O_2_ by taking Spm, T-Spm, and Nor-Spm as substrates to facilitate PA metabolism. It will be interesting to further investigate the substrate preference of CaPAO1.

The other source of apoplastic ROS during EBL-induced AR formation is PM-NADPH oxidase (CaRBOHs). The roles of RBOH-derived O_2_^•−^ in AR formation have been reported in many plant species, including Arabidopsis, cucumber, mung bean, apple, poplar, and so on [23,32,33,77]. In the present study, we found that CaRBOH-dependent O_2_^•−^ contributed to EBL-induced AR formation in pepper. Of the *CaRBOH* gene family, *CaRBOH2*, *CaRBOH5*, and *CaRBOH6* were induced by EBL treatment, but repressed in response to BRz application (Figure 6). Co-application with DPI and silencing these genes via the VIGS system compromised EBL-induced O_2_^•−^ production and halted the promoting effects of EBL on AR formation (Figure 4 and Figure 8). Of these involved CaRBOH proteins, CaRBOH2 was found in the same clade as AtRBOHD, which has been found to be involved in mediating AR formation in Arabidopsis hypocotyl cuttings, where AtRBOHD participated in the upward propagation of ROS from the cutting base in the intact hypocotyls after wounding [24]. CaRBOH5 and AtRBOHE belong to the same clade. Although the roles of AtRBOHE in AR formation have not been reported, it has been found that AtRBOHE plays important roles in lateral root formation [78]. *AtRBOHE* was expressed in both lateral root primordia and the overlaying epidermal cells, and modulated lateral root primordia initiation and promoted the cell wall remodeling of overlying parental tissues to help lateral root emergence [78]. In pepper, two expression peaks (72 h and 192 h) were observed for *CaRBOH5* during EBL-induced AR formation (Figure 6), which implied similar roles of CaRBOH5 in AR formation and AtRBOHE in lateral root formation. For CaRBOH6, its expression showed a substantial increase until 192 h (Figure 6), indicating that CaRBOH6 might also be involved in AR emergence and elongation. Similar cell-elongation-promoting roles were also found for AtRBOHH and AtRBOHJ [79], the homologs of CaRBOH6. Consistently, the silencing of *CaRBOH6* by VIGS resulted in a shorter AR phenotype in pepper (Figure 8). Collectively, these results indicated that CaRBOHs-derived O_2_^•−^ participated in both EBL-induced AR initiation and elongation.

In plants, PAOs and RBOHs are functionally interlinked in controlled ROS production and homeostasis; however, their coordination is controversial [26]. Gémes et al. presented a feedforward loop involving apoplastic PAOs and RBOHs in ROS accumulation in tobacco under salt stress [80]. In this loop, NaCl exposure induced RBOHs to produce O_2_^•−^, and then O_2_^•−^ activated the apoplastic PAOs to amplify H_2_O_2_ accumulation. Then, PAO-generated H_2_O_2_ could open Ca^2+^ channels and thereby increase the activity of the Ca^2+^-regulated RBOH enzymes and form a RBOH-PAO amplification loop. Finally, this RBOH-PAO loop caused an ROS accumulation that surpassed a toxicity threshold, resulting in programmed cell death [80]. However, a negative effect of PAO action on RBOH was reported in Arabidopsis, where mutations of the *AtPAO1* and *AtPAO2* genes resulted in enhanced O_2_^•−^ generation and RBOH activity [81]. In this study, during EBL-induced AR formation in pepper, whether and how PAO and RBOHs coordinated with each other requires further refinement.

## 5. Conclusions

**Figure 9:**
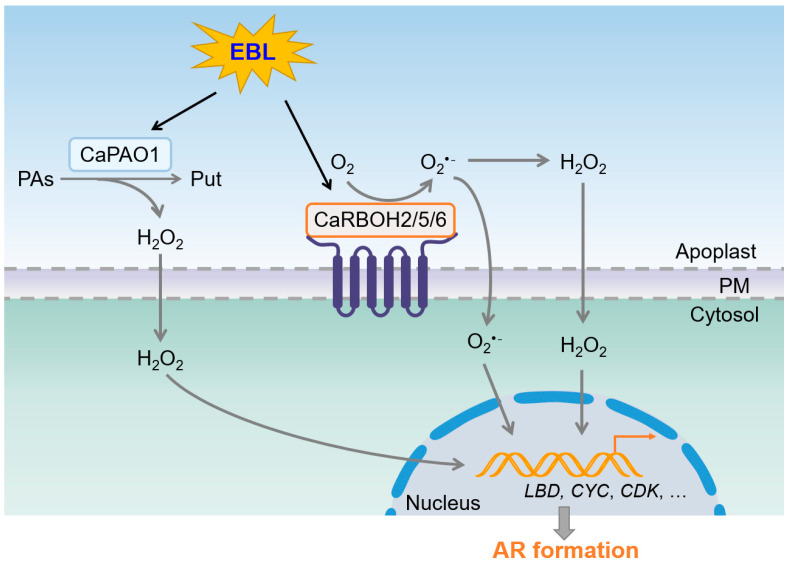
Schematic model for EBL-induced AR formation by coordinating ROS generation in pepper. EBL induces CaPAO1-derived H_2_O_2_ generation and CaRBOH2/5/6-derived O_2_^•−^ generation in apoplasts. O_2_^•−^ and H_2_O_2_ trigger signaling transduction to regulate the expression of *CaLBD*, *CaCYC*, and *CaCDK* genes, and further stimulate AR formation. PAs, polyamines. Put, putrescine. PM, plasma membrane.

Taken together, we propose a model for ROS generation during EBL-induced AR formation in pepper hypocotyls (Figure 9). EBL stimulates the expression of *CaPAO1* to increase apoplastic H_2_O_2_ generation. EBL also induces the expression of *CaRBOH2*, *CaRBOH5*, and *CaRBOH6* to increase apoplastic O_2_^•−^ generation. Then, O_2_^•−^ can be transformed to H_2_O_2_. These ROS signals enter the cytoplasm and trigger signaling transduction, which further regulates the expression of *LBD* genes and cell cycle genes to initiate AR formation and promote AR elongation. This model helps elucidate the regulatory mechanism underlying EBL-induced AR formation. In future studies, the detailed signaling cassette behind ROS-induced AR formation needs to be identified.

## Data Availability

Data are contained within the article and Supplementary Materials.

## Supplementary Materials

The following supporting information can be downloaded at https://www.mdpi.com/article/10.3390/antiox12071451/s1, Table S1: Information on *PAO* and *RBOH* genes for phylogenetic analysis. Table S2: List of primers for qRT-PCR in this study. Table S3: List of primers for plasmid construction in this study. Table S4: List of genes involved in pepper AR formation. Figure S1: Effects of KI, DMTU, MDL72527, 2-HEH, or DPI on AR formation in pepper hypocotyls. Figure S2: Effects of EBL on the activities of cytoplasmic PAO (CP-PAO) during AR formation in pepper hypocotyls. Figure S3: Phylogenetic relationship, functional domains, and gene structure of *CaPAO*s. (A) Phylogenetic relationship among gene structures of *CaPAO*s. (B) Distribution of conserved functional domains in CaPAO proteins. Figure S4: Phylogenetic relationship, functional domains, and gene structure of *CaRBOHs*. (A) Phylogenetic relationship among gene structures of *CaRBOH*s. (B) Distribution of conserved functional domains in CaRBOH proteins. Figure S5: Domain structure and sequence alignment of CaRBOH proteins. (A) Schematic representation of the CaRBOH proteins with the respective functional domains, including EF-hands, transmembrane domains (TMs), and flavin adenine dinucleotide (FAD)-binding sites. (B) Sequence alignment of seven CaRBOH proteins. EF-hand domains are indicated by orange boxes. Conserved FAD-binding sites are indicated by green box. Putative transmembrane domains predicted by TMHMM2.0 (https://services.healthtech.dtu.dk/service.php?TMHMM-2.0, accessed on 15 March 2023) are indicated by blue boxes. Figure S6: Phenotypes of the VIGS seedlings. (A) The top and side view of the control (*TRV2:00*) and PDS-silenced pepper plants (*TRV2:PDS*). (B) The side view of *CaPAO1*- and *CaRBOH*-silenced pepper plants.
