# Supplementary material for: 24-Epibrassinolide Facilitates Adventitious Root Formation by Coordinating Cell-Wall Polyamine Oxidase- and Plasma Membrane Respiratory Burst Oxidase Homologue-Derived Reactive Oxygen Species in Capsicum annuum L
Source: Antioxidants (Basel). 2023 Jul 19;12(7):1451. doi: 10.3390/antiox12071451 (PMC10376213; doi:10.3390/antiox12071451)
Supplement: Supplementary file 1 [file antioxidants-12-01451-s001.zip › antioxidants-2483594-supplementary.pdf]

Supplementary materials

Table S1. Information of *PAO* and *RBOH* genes for phylogenetic analysis. Different lowercase letters indicated that the values were significantly different among different treatments ($p < 0.05$).

Table S2. List of primers for qRT-PCR in this study.

Table S3. List of primers for plasmid construction in this study.

Table S4. List of genes involved in pepper AR formation.

Figure S1. Effects of KI, DMTU, MDL72527, 2-HEH or DPI on AR formation in pepper hypocotyls.

Figure S2. Effects of EBL on the activities of cytoplasmic PAO (CP-PAO) during AR formation in pepper hypocotyls.

Figure S3. Phylogenetic relationship, functional domains, and gene structure of *CaPAOs*. **(A)** Phylogenetic relationship among gene structures of *CaPAO*s. **(B)** Distribution of conserved functional domains in CaPAO proteins.

Figure S4. Phylogenetic relationship, functional domains, and gene structure of *CaRBOHs*. **(A)** Phylogenetic relationship among gene structures of *CaRBOH*s. **(B)** Distribution of conserved functional domains in CaRBOH proteins.

Figure S5. Domain structure and sequence alignment of CaRBOH proteins. **(A)** Schematic representation of the CaRBOH proteins with the respective functional domains, including EF-hands, transmembrane domains (TMs) and flavin adenine dinucleotide (FAD)-binding sites. **(B)** Sequence alignment of seven CaRBOH proteins. EF-hand domains are indicated by orange boxes. Conserved FAD-binding site are indicated by green box. Putative transmembrane domains predicted by TMHMM2.0 (https://services.healthtech.dtu.dk/service.php?TMHMM-2.0) are indicated by blue boxes.

Figure S6. Phenotypes of the VIGS seedlings. **(A)** The top- and side-view of the control (*TRV2:00*) and PDS-silenced pepper plants (*TRV2:PDS*). **(B)** The side-view of *CaPAO1*- and *CaRBOH*-silenced pepper plants.

Table S1. Information of *PAO* and *RBOH* genes for phylogenetic analysis

Species	*PAO* family		*RBOH* family	
	Gene name	Accession No.	Gene name	Accession No.
Arabidopsis thaliana	*AtPAO1*	At5g13700	*AtRBOHA*	At5g07390
	AtPAO2	At2g43020	*AtRBOHB*	At1g09090
	AtPAO3	At3g59050	*AtRBOHC*	At5g51060
	AtPAO4	At1g65840	*AtRBOHD*	At5g47910
	AtPAO5	At4g29720	*AtRBOHE*	At1g19230
	AtPAO6	At1g62830	*AtRBOHF*	At1g64060
	AtPAO7	At3g13682	*AtRBOHG*	At4g25090
	AtPAO8	At4g16310	*AtRBOHH*	At5g60010
	AtPAO9	At3g10390	*AtRBOHI*	At4g11230
			AtRBOHJ	At3g45810
Solanum lycopersicum	*SlPAO1*	Solyc01g087590	*SlRBOH1*	Solyc06g068680
	SlPAO2	Solyc07g043590	*SlRBOH2*	Solyc03g117980
	SlPAO3	Solyc12g006370	*SlRBOH3*	Solyc01g099620
	SlPAO4	Solyc02g081390	*SlRBOH4*	Solyc05g025680
	SlPAO5	Solyc03g031880	*SlRBOH5*	Solyc07g042460
	SlPAO6	Solyc05g018880	*SlRBOH6*	Solyc06g075570
	SlPAO7	Solyc07g039310	*SlRBOH7*	Solyc11g072800
	SlPAO8	Solyc12g008850	*SlRBOH8*	Solyc08g081690
	SlPAO9	Solyc07g063450		
	SlPAO10	Solyc07g063500		
	SlPAO11	Solyc04g081100		
Capsicum annuum	*CaPAO1*	LOC107858653	*CaRBOH1*	LOC107875997
	CaPAO2	LOC107878076	*CaRBOH2*	LOC107862088
	CaPAO3	LOC107841896	*CaRBOH3*	LOC107839607
	CaPAO4	LOC107859655	*CaRBOH5*	LOC107877834
	CaPAO5	LOC107870798	*CaRBOH6*	LOC107875569
	CaPAO8	LOC107840963	*CaRBOH7*	LOC107846897
	CaPAO10	LOC107878694	*CaRBOH8*	LOC107864242
	CaPAO11	LOC107866910		

Table S2. List of primers for qRT-PCR in this study.

Primer name	Primer sequences (5'-3')
qCaPAO1-F	AGTGGAATAGCAGCGGACTC
qCaPAO1-R	CGTTTTTGGTGTACTGGCTGG
qCaPAO2-F	GCTGAGGCTTCACCATCTGT
qCaPAO2-R	GCTGAGGCTTCACCATCTGT
qCaPAO3-F	GGAAGGAGGTCTGTGGCAAA
qCaPAO3-R	GCATCATGAAGTGTGCGAGC
qCaPAO4-F	GAGTTGAGGCAAGAAGGGCT
qCaPAO4-R	CATGGCCACCAGTGAGAACT
qCaPAO5-F	GGCAGTCTGATGACCACCAA
qCaPAO5-R	TTTAGGACCCCAAGCGACAC
qCaPAO8-F	GACGCGAAGTTGAACAACCC
qCaPAO8-R	TTGTTGTCGAGACCCCTTCG
qCaRBOH1-F	TTGTGTTTGTGTCGCCAAGG
qCaRBOH1-R	GCCACCGCCATCATTTTGTG
qCaRBOH2-F	TGTTTGTCAACTGTGCTGCG
qCaRBOH2-R	GGTGGTTGGCAAACCTCAGA
qCaRBOH3-F	AGGCGATTGGACATCTCAGC
qCaRBOH3-R	AGAGCTTTGGCAATCTAGGCT
qCaRBOH5-F	TCTGGCAGTGGTGTATGTGC
qCaRBOH5-R	TCCTGGAAGTACAGAAACCTTCA
qCaRBOH6-F	GCAGTCTCCCCACTAGAACC
qCaRBOH6-R	TCGCCCTCTTTCCCTGTAGT
qCaRBOH7-F	GGGTTCAAGAGCCTGCATTT
qCaRBOH7-R	CAAATTCCTTGCTTTCCATTCCTAT
qCaRBOH8-F	TGCAACAAGCGAACAACCAG
qCaRBOH8-R	TCTCGATGACTCCCCGTTGA
qCaLBD16-F	CCGTTTCCTAGTCTGCTCGT
qCaLBD16-R	TTGCATGCACCACAAGGTGA
qCaLBD29-F	AGCAGCAGCTCAAAACTTGC
qCaLBD29-R	TGGGGCATAGTGCTTGGATT
qCaCYCD3;1-F	GTCATGTGGTGGGTAGCACT
qCaCYCD3;1-R	TGCAATCCACCAGCTAACCA
qCaCDKA1-F	CATACTCAAAGAGTCCATCCAGC
qCaCDKA1-R	TCCACGATCACGAGCTTTGT
qCaCDKD3-F	GGCCGCTGCATGTATCTTTG
qCaCDKD3-R	ACTGTGAAGGCTTTGGGGTC
qCaUBI3-F	TGTCCATCTGCTCTCTGTTG
qCaUBI3-R	CACCCCAAGCACAATAAGAC

Table S3. List of primers for plasmid construction in this study.

Primer name	Primer sequences (5'-3')
**1. Construction of VIGS vectors**	
TRV2-CaPAO1-F	gtgagtaaggttaccgaattcAATATCTTCCCACTGTGATCATAGATG
TRV2-CaPAO1-R	cgtgagctcggtaccggatccTCGCTCCTCTGTAATCATCGTCG
TRV2-CaRBOH2-F	gtgagtaaggttaccgaattcTTTAGCCAATAAAGCCAACTCCG
TRV2-CaRBOH2-R	cgtgagctcggtaccggatccATGCAGAATTCGGAAAATCATCA
TRV2-CaRBOH5-F	gtgagtaaggttaccgaattcAGCTTCTTCTTCGCCTTCATCA
TRV2-CaRBOH5-R	cgtgagctcggtaccggatccATGCAGCTGAAATCATTCTTGAGG
TRV2-CaRBOH6-F	gtgagtaaggttaccgaattcATTCGTGGTACAACTCCACTCCTT
TRV2-CaRBOH6-R	cgtgagctcggtaccggatccGCACTATGGTTTTTAAAAACGTTGTAT
**2. Construction of transient expression vectors**	
2300-CaPAO1-F	tatgaccatgattacgaattcATGCAGAATTCGGAAAATCATCA
2300-CaPAO1-R	caggtcgactctagaggatccTCAAAAATTTTCTTTATGGAAATCAAA
2300-CaRBOH2-F	tatgaccatgattacgaattcATGCAGAATTCGGAAAATCATCA
2300-CaRBOH2-R	caggtcgactctagaggatccTCAAAAATTTTCTTTATGGAAATCAAA
2300-CaRBOH5-F	tatgaccatgattacgaattcATGCAGCTGAAATCATTCTTGAGG
2300-CaRBOH5-R	caggtcgactctagaggatccTCATACCCGAGTGCCAGACAG
2300-CaRBOH6-F	tatgaccatgattacgaattcATGGTGCCCATTGACAATGG
2300-CaRBOH6-R	caggtcgactctagaggatccCTAGAAATTTTCTTTGTGGAAATTGAA

Table S4. List of genes involved in pepper AR formation

Gene name	Accession No.
CaLBD29	LOC107853240
CaLBD16	LOC107853239
CaCYCD3;1	LOC107840237
CaCDKA-1	LOC107850646
CaCDKD-3	LOC107864765

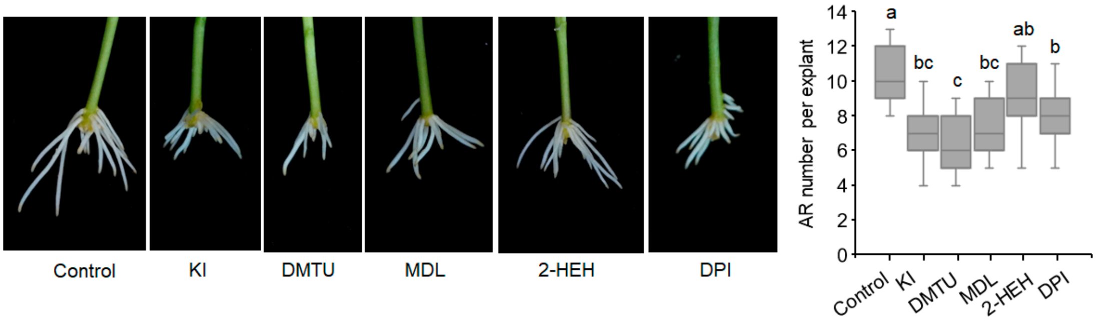

Figure S1. Effects of KI, DMTU, MDL72527, 2-HEH or DPI on AR formation in pepper hypocotyls. Different lowercase letters indicated that the values were significantly different among different treatments ($p < 0.05$).

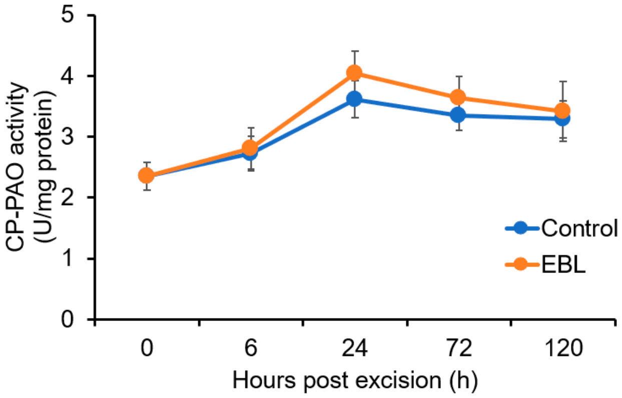

Figure S2. Effects of EBL on the activities of cytoplasmic PAO (CP-PAO) during AR formation in pepper hypocotyls.

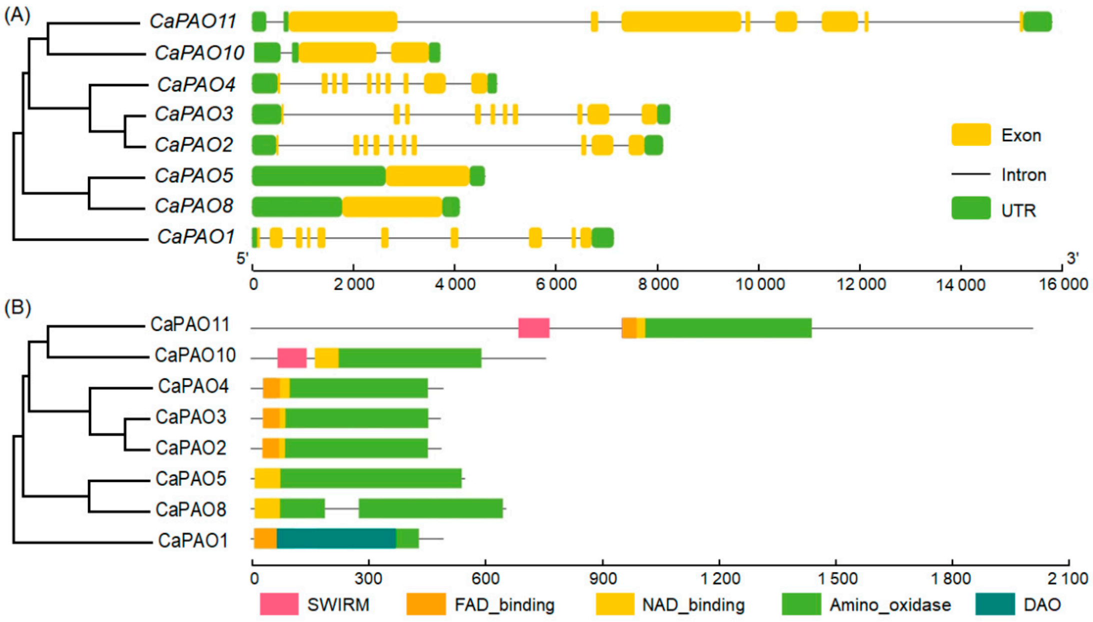

Figure S3. Phylogenetic relationship, functional domains, and gene structure of *CaPAOs*. **(A)** Phylogenetic relationship among gene structures of *CaPAO*s. **(B)** Distribution of conserved functional domains in CaPAO proteins.

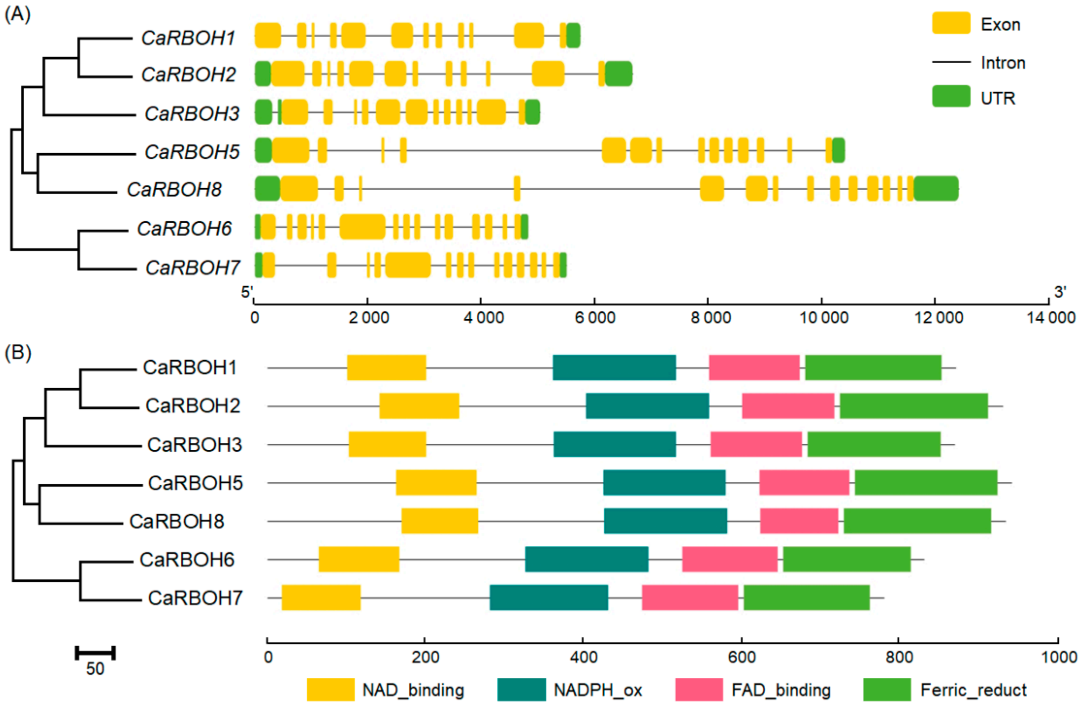

Figure S4. Phylogenetic relationship, functional domains, and gene structure of *CaRBOHs*. **(A)** Phylogenetic relationship among gene structures of *CaRBOH*s. **(B)** Distribution of conserved functional domains in CaRBOH proteins.

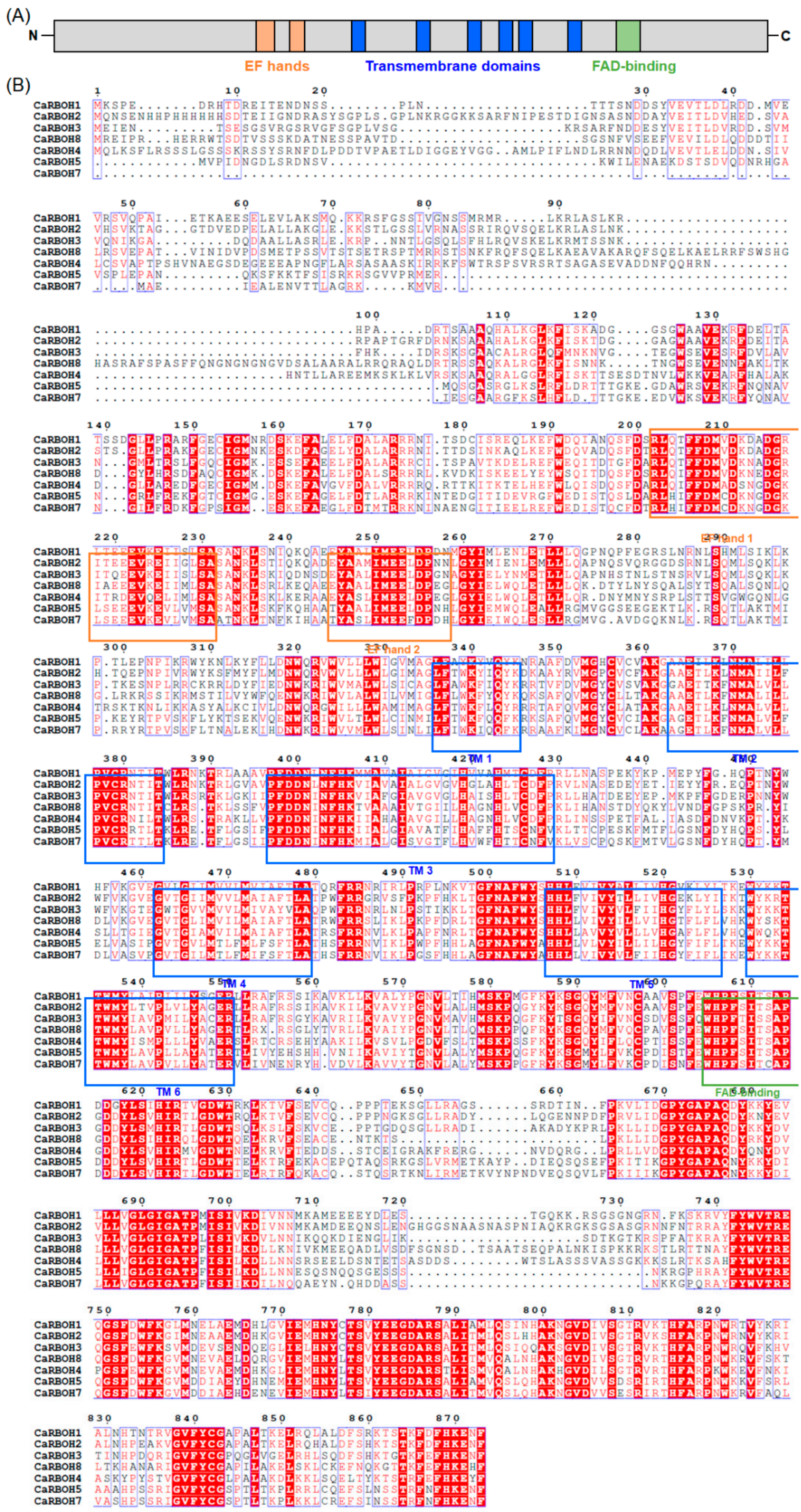

Figure S5. Domain structure and sequence alignment of CaRBOH proteins. **(A)** Schematic representation of the CaRBOH proteins with the respective functional domains, including EF-hands, transmembrane domains (TMs) and flavin adenine dinucleotide (FAD)-binding sites. **(B)** Sequence alignment of seven CaRBOH proteins. EF-hand domains are indicated by orange boxes. Conserved FAD-binding site are indicated by green box. Putative transmembrane domains predicted by TMHMM2.0 (https://services.healthtech.dtu.dk/service.php?TMHMM-2.0, accessed on 15 March 2023) are indicated by blue boxes.

(A)

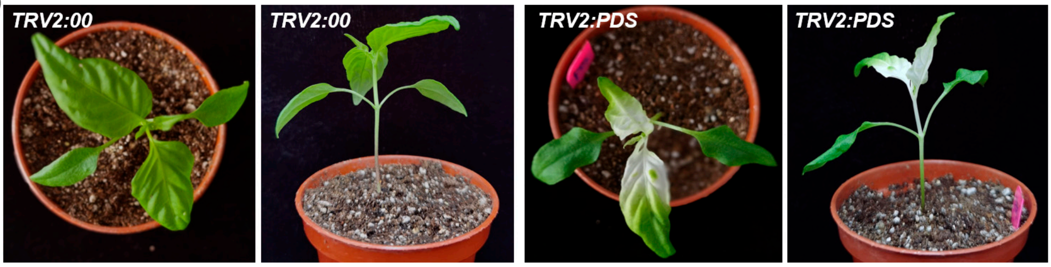

(B)

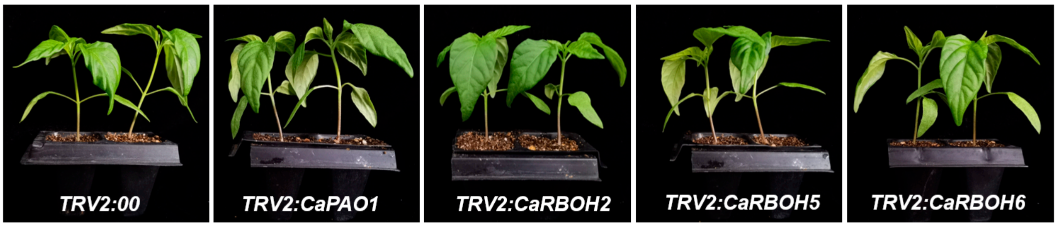

Figure S6. Phenotypes of the VIGS seedlings. **(A)** The top- and side-view of the control (*TRV2:00*) and PDS-silenced pepper plants (*TRV2:PDS*). **(B)** The side-view of *CaPAO1*- and *CaRBOH*-silenced pepper plants.
